# *EGFR*突变的肺腺癌患者中Rb和磷酸化Rb的表达及临床特征分析

**DOI:** 10.3779/j.issn.1009-3419.2017.05.03

**Published:** 2017-05-20

**Authors:** 云龙 董, 明辉 刘, 永文 李, 颖 李, 辰龙 赵, 茵 袁, 晖 杜, 子禾 张, 洪兵 张, 红雨 刘, 军 陈

**Affiliations:** 1 300052 天津，天津医科大学总医院肺部肿瘤外科 Department of Lung Cancer Surgery; 2 300052 天津天津市肺癌研究所，天津市肺癌转移与肿瘤微环境实验室 Tianjin Key Laboratory of Lung Cancer Metastasis and Tumor Microenvironment, Tianjin Lung Cancer Institute, Tianjin Medical University General Hospital, Tianjin 300052, China

**Keywords:** 肺肿瘤, Rb, pRb-780, pRb-795, *EGFR*, Lung neoplasms, Rb, pRb-780, pRb-795, *EGFR*

## Abstract

**背景与目的:**

Rb作为重要的抑癌基因，调控细胞周期的进程。各种原因导致的Rb功能异常均可导致细胞的持续过度增殖从而导致肿瘤的发生。Rb蛋白表达缺失或减弱及过度磷酸化是Rb功能异常的重要机制。具有突变的表皮生长因子受体（epidermal growth factor receptor, EGFR）基因是肺腺癌重要的驱动基因，在肺癌的发生发展中起着重要的作用。本研究目的在于探讨Rb在EGFR突变的肺腺癌中的存在状态。

**方法:**

取23例具有EGFR突变的肺腺癌标本，用免疫组化的方法分析Rb、pRb-780、pRb-795表达状态及临床特征。

**结果:**

在23例EGFR突变的肺腺癌患者中Rb蛋白表达缺失/减弱频率为69.6%，pRb-780、pRb-795过表达的频率分别为73.9%、69.6%。23例患者均存在Rb表达缺失/减弱或Rb过度磷酸化。进一步分析发现，pRb-780过表达在晚期患者中发生更多（*P*=0.022）；pRb-795过表达在晚期患者中发生更多，但无统计学差异（*P*=0.074）。

**结论:**

在EGFR突变的肺腺癌患者中，频繁发生Rb的表达缺失/减弱或过度磷酸化，Rb功能异常是EGFR突变肺腺癌患者重要的发病机制。

## Abstract (en)

**Background and objective:**

Rb is an important tumor suppressor gene that regulates cell cycle progression. Rb dysfunction can lead to over proliferation of cells and lead to the occurrence of tumor. Loss or reduced Rb expression as well as over phosphorylation of Rb are important mechanisms of Rb dysfunction. The mutated epidermal growth factor receptor (*EGFR*) gene is an important driver gene in lung adenocarcinoma, and plays an important role in the development of lung cancer. The purpose of this study was to investigate the status of Rb in lung adenocarcinoma patients with *EGFR* mutations and define the clinicopathologic features.

**Methods:**

23 cases pulmonary adenocarcinoma patients with *EGFR* mutations were collected. The status of Rb and pRb-780, pRb-795 were determined byimmunohistochemistry.

**Results:**

Loss or reduced Rb expression were detected in 16 of 23 samples (69.6%). pRb-780 and pRb-795 over-expressed were identified in 17 (73.9%) and 16 (69.6%) of 23 samples respectively. All the 23 patients had showed loss/reduced Rb expression or over-expressed Rb phosphorylation. Further analysis showed that over expression of pRb-780 and pRb-795 occurred more frequently in advanced patients.

**Conclusion:**

Aberrant expressionof Rb is frequently occurred in lung adenocarcinoma patients with *EGFR* mutations and may be an important pathogenesis in patients with lung adenocarcinoma.

肺癌已经跃居肿瘤发病率之首，成为癌症死亡的主要原因。肺癌发病的根本原因，在于体内原癌基因的激活或者抑癌基因的失活，致使细胞恶性增殖及不可逆转化，最终导致癌症的发生^[1-4]^。RB基因（视网膜细胞瘤基因）是人体内重要的抑癌基因，通过控制细胞周期的进程，抑制细胞的过度增殖。Rb-E2F信号通路是调控细胞周期G1后期通过限速点R到S期的关键信号分子和通路，通常情况下，Rb与E2F1结合，使E2F1没有活性，但在肿瘤中经常由于突变、杂合性缺失（loss of heterozygosity, LOH）、甲基化或磷酸化等原因使Rb失活，使之释放出与之结合的转录因子E2F1，E2F1可促进cyclin E、cyclin A等能控制细胞分裂的基因的转录，使细胞周期进展，细胞增殖^[5-11]^。多项研究已经证实，在肺癌中普遍存在Rb蛋白的表达异常。比如，有研究显示非小细胞肺癌（non-small cell lung cancer, NSCLC）中约20%-40%存在Rb蛋白表达降低。另有研究提示，NSCLC中约12.5%的患者存在Rb的表达降低，在不同的肺癌类型中，表达情况不同，其中鳞癌中约有5.5%，肺腺癌中有19.4%。其他研究显示在大细胞肺癌、小细胞肺癌的突变率较高，约68%和87%^[12-14]^。因此Rb的异常普遍存在于肺癌中，是肺癌重要的发病因素。

表皮生长因子受体（epidermal growth factor receptor, EGFR）基因作为肺腺癌重要的驱动基因（diver gene），在肺癌的发生发展中起着重要的作用。EGFR信号通路一旦被激活，可导致肿瘤细胞内酪氨酸蛋白激酶活化和受体自身磷酸化，从而促使细胞增生、分化、转移、血管生成及凋亡抑制。EGFR酪氨酸激酶抑制剂（EGFRTKI）通过阻断细胞受体的三磷酸腺苷结合位点，阻止下游信号传递而产生肿瘤抑制作用。在具有EGFR突变的NSCLC患者中，使用EGFR-TKI疗效优于传统的化疗，且毒副作用小，患者耐受性好。EGFR外显子19或/和21的突变异常将导致EGFR结合ATP的能力增加，从而加强了TKI的疗效因此，具有EGFR突变的NSCLC患者是EGFRTKI治疗的优势人群，而女性、非吸烟的腺癌患者，其EGFR突变发生频率更高，成为EGFR-TKI治疗的优势人群^[15-20]^。

在*EGFR*突变的肺腺癌患者中Rb及磷酸化Rb的表达情况未见文献报道。本研究将利用免疫组化技术，分析23例具有*EGFR*突变的肺腺癌患者肿瘤组织中Rb及磷酸化pRb-780、pRb-795的表达情况，分析在具有*EGFR*突变的患者中Rb的异常表达与患者临床病理生理特征的关系以及与患者预后的关系，探讨EGFR信号通路与Rb信号通路是否具有协同性。

## 1 材料与方法

### 1.1 患者的基本信息以及临床资料

23例EGFR突变的肺腺癌患者标本取自天津医科大学总医院肺部肿瘤外科2014年1月-2015年12月手术患者，术后病理诊断为肺腺癌。术前未接受放疗、化疗及分子靶向治疗。EGFR突变检测分别由广州益善公司和广州燃石公司完成。表 1列出了23例患者的详细临床信息。共有14例男性，9例女性，年龄26岁-71岁，中位年龄62岁，吸烟者11例，不吸烟12例，临床Ⅰ期/Ⅱ期10例，Ⅲ期/ Ⅳ期13例；EGFR 19外显子突变患者11例，21外显子突变9例，20外显子突变2例，其他位点突变有1例（表 1）。

**1:** 患者的统计资料 Patient demographics

Category	Patients
Age, median (range)	62 (26-71)
< 62	11 (47.8%)
≥62	12 (52.2%)
Gender	
Male	14 (60.8%)
Female	9 (39.1%)
Smoker	
Yes	11 (47.8%)
No	12 (52.2%)
Clinical stage	
Ⅰ/Ⅱ	10 (43.5%）
Ⅲ/Ⅳ	13 (56.5%）
Tumor location	
Left	9 (39.1%)
Right	13 (56.5%)
Both	1 (4.4%)
*EGFR* mutations	
19	11 (47.8%)
21	9 (39.1%)
20	2 (8.7%)
Other	1 (4.4%)
EGFR: epidermal growth factor receptor.	

### 1.2 免疫组化

23例组织标本均由天津医科大学总医院病理科常规固定、包埋，切片。4 μm-6 μm厚的常规病理石蜡切片，70 ℃烤片40 min，脱蜡，脱水后，经Tris/EDTA修复液（pH8.0）进行抗原修复，正常羊血清RT封闭30 min，加入一抗4 ℃过夜，经生物素化的二抗37 ℃ 30 min，辣根酶标记的链霉素卵白素工作液37 ℃ 30 min，DAB显色液显色，苏木素复染。抗体稀释倍数为1:20-1:100。Rb、pRb-780、pRb-795兔抗人多克隆抗体均购自Cell Signaling Technology公司。

Rb、pRb-780、pRb-795阳性染色呈棕黄色或者黄褐色颗粒，三种蛋白主要在细胞核中表达，由于Rb基因的杂合缺失可以导致正常Rb蛋白表达缺失或者表达一种不完全的Rb蛋白，这一部分的Rb蛋白不进入细胞核，所以可能导致细胞质的轻度着色，所以在免疫组化染色中会出现一部分在细胞质染色的病例，我们将其划归为非特异染色^[12, 21]^。Rb的结果判定：根据在400×高倍镜下比较23例肺癌组织标本和同一例患者癌旁肺组织的阳性染色程度，对阳性染色的癌组织以及癌旁肺组织的阳性染色进行评分，若肺癌组织中染色强度比癌旁肺组织强或者相同，则判断为无表达降低/缺失，若癌组织染色比癌旁肺组织染色弱或无，即判定为Rb蛋白表达降低或缺失。pRb的判定：根据400×高倍镜下，若肺癌组织中阳性染色强度比癌旁肺组织高，则判断为Rb过度磷酸化，若癌组织染色与癌旁肺组织染色相同或弱，即判定无Rb过度磷酸化。切片不加入一抗，作为空白对照。

### 1.3 统计学分析

统计软件采用SPSS 20.0进行数据分析，用卡方检验及非参数检验，生存分析采用*Kaplan-Meier*法，以*P* < 0.05为具有统计学意义。

## 2 结果

### 2.1 Rb及pRb-780、pRb-795在*EGFR*突变肺腺癌患者中的表达情况

Rb蛋白主要在细胞核中表达，一部分病例阳性染色细胞排列呈巢状，周围被结蹄组织分割，另可见部分患者的细胞排列成腺腔样（图 1）。在23例肺腺癌患者中，16例（16/23, 69.6%）存在表达降低/缺失。Rb的表达情况与患者临床病理生理特征分析后发现，在不同年龄、性别、吸烟史、临床分期、肿瘤位置及不同的EGFR突变位点的患者中未见显著性差异。

**1:**
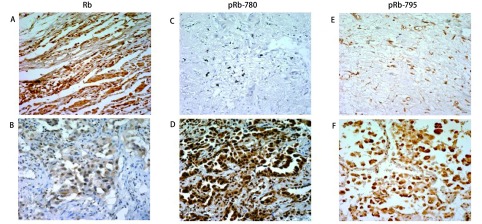
Rb（A、B）及pRb-780（C、D）、pRb-795（E、F）免疫组化结果示意图。Rb在肺腺癌组织细胞（B）中较癌旁肺组织（A）中表达减低；pRb-780和pRb-795在肺腺癌组织（D、F）中比癌旁肺组织（C、E）中过表达。 Representative Rb (A, B), pRb-780 (C, D), pRb-795 (E, F) immunostaining results. Reduced expression of Rb in adenocarcinoma cells were detected (B) compared to adjacent lung tissue cells (A). pRb-780 and pRb-795 were overexpressed in adenocarcinoma (D, F) compared to adjacent lung tissue cells (C, E).

pRb-780表达与Rb类似，表达于细胞核中，大部分阳性肿瘤细胞排列成腺腔样；可见部分排列成巢状，呈鳞状分化，周围可被纤维组织分隔；少数的病例阳性表达的肿瘤细胞散在分布。在同一病例中可见不规则分布的从低到高度的表达差异。23例患者中有17例患者存在pRb-780染色增强，即pRb-780过表达，占73.9%。分析患者例临床信息与pRb-780蛋白表达的关系发现，Ⅲ期/Ⅳ期患者中与Ⅰ期/Ⅱ期患者相比，pRb-780蛋白过表达率更高，差异有统计学意义（*P*=0.022）；未发现与年龄、性别、吸烟史、肿瘤位置等临床信息相关。

pRb-795同样主要在细胞核中表达，阳性肿瘤细胞排列成巢状、腺腔样、偶尔可见呈乳头状排列。16例（16/23, 69.6%）患者存在pRb-795的过表达。通过分析pRb-795蛋白的表达情况与患者临床病理生理特征发现：与Ⅰ期/Ⅱ期患者相比，Ⅲ期/Ⅳ期有更多的患者存在pRb-795的过表达，但是差异不显著（*P*=0.074）；其他未见明显差异（表 2）。

综合分析发现，23例患者均存在Rb表达降低和/或pRb增加。

**2:** 23例肺癌患者中Rb and p-Rb的表达情况以及临床信息 The expression of Rb and p-Rb in 23 lung cancer patients by immunohistochemical staining and their clinical characteristics

Factor		Rb				pRb-780				pRb-795		
		Reduced	Non-reduced	*P*		Non-overexpressed	Over-expressed	*P*		Non-overexpressed	Over-expressed	*P*
*n*=23	100%	16 (69.6%)	7 (30.4%)			6 (26.1%)	17 (73.9%)			7 (30.4%)	16 (69.6%)	
Age				0.752				0.408				0.221
< 62	11 (47.8%)	8	3			2	9			2	9	
≥62	12 (52.2%)	8	4			4	8			5	7	
Gender				0.809				0.190				0.242
Male	14 (60.8%)	10	4			5	9			3	11	
Female	9 (39.1%)	6	3			1	8			4	5	
Smoking history				0.221				0.901				0.752
Yes	11 (47.8%)	9	2			3	8			3	8	
No	12 (52.2%)	7	5			3	9			4	8	
Clinical stage				0.340				0.022*				0.074
Ⅰ/Ⅱ	10 (43.5%)	8	2			5	5			5	5	
Ⅲ/Ⅳ	13 (56.5%)	8	5			1	12			2	11	
Tumor location				0.448				0.752				0.789
Left	9 (39.1%)	5	4			2	7			3	6	
Right	13 (56.5%)	10	3			4	9			4	9	
Both	1 (4.4%)	1	0			0	1			0	1	
*EGFR* mutations				0.811				0.613				0.122
19	11 (47.8%)	8	3			2	9			2	9	
21	9 (39.1%)	6	3			2	7			5	4	
20	2 (8.7%)	1	1			1	1			0	2	

### 2.2 Rb、pRb-780、pRb-795表达情况与*EGFR*突变肺腺癌患者的预后分析

在23例患者中，有20例具有完整的随访信息，其中，死亡3例，至今生存17例。我们进一步依据其Rb、pRb-780、pRb-795的表达情况进行了生存期的初步分析。在Rb表达无降低和表达降低的患者，生存时间分别为26个月和24.8个月；pRb-780表达增强和无增强的患者，生存时间分别为24.8个月和26.1个月，pRb-795表达增强和无增强的患者，生存时间分别为24.8个月和26.5个月；存在Rb表达异常的患者以及P-Rb过表达的患者生存时间短，但是统计学未见差异（表 3）。

**3:** 23例肺癌患者的生存时间 The survival time of 23 lung cancer patients

Factor	*n*=23 (100%)	TIME	*P*
Rb			0.662
Reduced	16 (70%)	24.8	
Non-reduced	7 (30%)	26	
pRb-780			0.46
Non-overexpressed	6 (26%)	26.1	
Over-expressed	17 (74%)	24.8	
pRb-795			0.888
Non-overexpressed	7 (30%)	26.5	
Over-expressed	16 (70%)	24.8	

## 3 讨论

Rb属于“袋蛋白”（Pocket Protein）家族，在细胞周期的调控中起到关键作用^[22]^。各种原因导致的Rb异常及功能缺失均可导致细胞周期及细胞增殖异常。而LOH导致的Rb表达降低以及各种原因导致的Rb过度磷酸化是Rb失活的重要机制^[23]^。如Witkiewicz报道^[24]^称在乳腺癌的发生发展中，Rb的失活是由许多相互协调的机制导致的，约50%的乳腺癌病人中可见cyclin D1的过表达，导致了Rb的磷酸化而失去功能；20-30%的乳腺癌患者中可见Rb基因的丢失；还有部分患者是由于P16的失活导致了Rb的磷酸化失活。为了探讨在具有*EGFR*突变的患者中Rb的存在状态，我们选用23例有*EGFR*突变的肺腺癌标本，应用免疫组化分别检测了Rb、pRb-780和pRb-795位点的蛋白表达情况，发现Rb的异常表达普遍存在于*EGFR*突变的肺腺癌患者中：约70%的患者存在Rb的表达降低/缺失，约70%的患者存在Rb的过度磷酸化，而100%的患者存在Rb的低表达或过度磷酸化，提示在*EGFR*突变的肺腺癌患者中，普遍存在Rb异常所导致的细胞周期异常。细胞周期异常是*EGFR*突变肺腺癌患者重要的发病机制。进一步可在细胞和动物模型水平探讨使用细胞周期抑制剂是否能有效抑制*EGFR*突变细胞的恶性增殖，将会是一个有益的尝试。

以往文献报道在NSCLC中，采用免疫组化方法检测到的Rb表达减弱或缺失的百分率为20%-40%左右，也有作者报道，在肺腺癌中，Rb表达减弱或缺失的频率高于鳞癌，而我们检测到在具有EGFR突变的肺腺癌患者中，Rb表达减弱或缺失的百分率为70%左右，远远高于文献报道的NSCLC患者中的百分率，是否提示在具有EGFR突变的肺腺癌中Rb的表达缺失/降低频率高于其他病例。由于本研究只采用了免疫组化的方法检测，需要进一步采取手段检测是否存在LOH、甲基化、突变等导致Rb表达减弱或缺失的机制。

本实验中23例*EGFR*突变的肺腺癌患者中Rb蛋白缺失或减弱以及Rb的过度磷酸化与患者的生存时间没有显著性差异，但是能看出，存在Rb蛋白缺失或减弱的以及存在Rb的高度磷酸化的患者生存时间偏短，推测是由于病例数偏少与随访时间不够造成的。
